# The interaction of TRPV1 and lipids: Insights into lipid metabolism

**DOI:** 10.3389/fphys.2022.1066023

**Published:** 2022-12-15

**Authors:** Shtaywy S. Abdalla, Amani A. Harb, Ihab M. Almasri, Yasser K. Bustanji

**Affiliations:** ^1^ Department of Biological Sciences, School of Science, The University of Jordan, Amman, Jordan; ^2^ Department of Basic Sciences, Faculty of Arts and Sciences, Al-Ahliyya Amman University, Amman, Jordan; ^3^ Department of Pharmaceutical Chemistry and Pharmacognosy, Faculty of Pharmacy, Al-Azhar University, Gaza, Palestine; ^4^ Department of Biopharmaceuticals and Clinical Pharmacy, School of Pharmacy, The University of Jordan, Amman, Jordan

**Keywords:** TRPV1, lipids, Ca^2+^, capsaicin, capsazepine, hyperlipidemia, uncoupling proteins

## Abstract

Transient receptor potential vanilloid 1 (TRPV1), a non-selective ligand-gated cation channel with high permeability for Ca^2+^, has received considerable attention as potential therapeutic target for the treatment of several disorders including pain, inflammation, and hyperlipidemia. In particular, TRPV1 regulates lipid metabolism by mechanisms that are not completely understood. Interestingly, TRPV1 and lipids regulate each other in a reciprocal and complex manner. This review surveyed the recent literature dealing with the role of TRPV1 in the hyperlipidemia-associated metabolic syndrome. Besides TRPV1 structure, molecular mechanisms underlying the regulatory effect of TRPV1 on lipid metabolism such as the involvement of uncoupling proteins (UCPs), ATP-binding cassette (ABC) transporters, peroxisome proliferation-activated receptors (PPAR), sterol responsive element binding protein (SREBP), and hypoxia have been discussed. Additionally, this review extends our understanding of the lipid-dependent modulation of TRPV1 activity through affecting both the gating and the expression of TRPV1. The regulatory role of different classes of lipids such as phosphatidylinositol (PI), cholesterol, estrogen, and oleoylethanolamide (OEA), on TRPV1 has also been addressed.

## 1 Introduction

Transient receptor potential vanilloid 1, or vanilloid receptor 1 (TRPV1/VR1), is a ligand-gated ion channel which belongs to the TRPV subfamily of the transient receptor potential (TRP) family. TRPV1 is a homotetrameric protein that has a wide extracellular “mouth” with a short selectivity filter. Each of the four subunits contains a highly variable N-terminal cytoplasmic region, followed by a ∼70-residue highly conserved linker region, a transmembrane domain comprised of six membrane-spanning α helices (S1-S6), a long pore-forming loop between S5 and S6, and a short cytoplasmic C-terminal region (Liao et al., 2013; Hellmich and Gaudet, 2014; Ramal-Sanchez et al., 2021).

TRPV1 cation channel is more selective to cations over anions but it poorly discriminates between small cations for its activation was found to generally exhibit higher permeability for Ca^2+^ over Na^+^ (PCa^2+^/PNa^+^ ≈ 3–10). The actual nature of this selectivity or permeability depends on a variety of factors, including the nature and the concentration of the agonist (Zhai et al., 2020). TRPV1 is mainly expressed in nervous system and its activation leads to the inflow of cations, resulting in a depolarization and action potentials in the afferent neurons which will propagate to the areas of the central nervous system responsible for heat and pain sensations (Zhang C. et al., 2020). TRPV1 is functionally involved in regulating Ca^2+^ homeostasis of organelles and cells. Its location on the plasma membrane allows passage of calcium ion from the extracellular fluid to the cytoplasm but when it is located intracellularly in various organelles such as the endoplasmic/sarcoplasmic reticulum and the mitochondria it helps controlling calcium level both inside the organelles and in the cytoplasm. Previous studies have shown that intracellularly located TRPV1 serves versatile functions in various physiological and pathological conditions (Zhao and Tsang, 2017). For example, activation of endoplasmic reticulum (ER)-localized TRPV1 is associated with ER stress and pro-apoptotic pathway (Zhao and Tsang, 2017; Zhai et al., 2020) whereas activation of mitochondrial TRPV1 is involved in mitochondrial Ca^2+^ uptake, leading to mitochondrial depolarization and contributing to cell migration, and its presence in Golgi apparatus may indicate its contribution in protein trafficking in the secretory pathway (Zhao and Tsang, 2017).

TRPV1 is a polymodal molecular integrator that can transduce information gathered from a diverse set of ligands since it can be activated by nociceptive thermal stimuli (temperature > 43°C), acidic environment (pH < 6.0), capsaicin and capsaicin analogues (e.g., resiniferatoxin), endogenous cannabinoids such as anandamide and over 15 additional endogenous lipids (Zhang C. et al., 2020; Zhai et al., 2020; Manchanda et al., 2021) whereas it is inhibited by capsazepine and ruthenium red. In addition, several other factors contribute to the regulation of TRPV1 gating including plasma membrane voltage change, protein phosphorylation by various protein kinases, previous activation of cannabinoid CB1 receptor by its lipophilic agonists, and direct or indirect regulation by some endogenous lipids, namely diacylglycerols and phosphatidylinositol-4,5-bisphosphate (PIP2) (De Petrocellis and Di Marzo, 2005).

The crucial role of TRPV1 in the conduction of pain, including heat and inflammatory pain was extensively studied. TRPV1 is found in the nervous system and in non-nerve tissues such as heart, liver, lung, kidney, adipose tissue, skeletal muscle, and intestine. Therefore, its involvement in many physiological processes such as thermoregulation, circadian rhythms, energy intake and lipid metabolism has been reported (Wang et al., 2011; Zhang C. et al., 2020; Zhai et al., 2020).

Recently, TRPV1 has become a research hotspot in metabolic disorder therapy. Several lines of evidence revealed the effective role of TRPV1 in lipid metabolism. González-Mercado et al. (2020) showed a possible relationship between TRPV1 gene polymorphisms and blood pressure and lipid profiles in a Mexican population. They showed that rs222747CC genotype, found in domain 5 containing ankyrin repeats, was associated with lower low density lipoprotein (LDL) levels, whereas rs224534AA genotype, located on the extracellular loop between helices 1 and 2, was associated with higher high density lipoprotein (HDL) levels and lower triglycerides and LDL (González-Mercado et al., 2020). Recently, the metabolic differences in plasma and skin between two female mice groups treated with or without capsaicin were studied. Using non-targeted metabolomics analysis, significant alterations in 38 plasma metabolites and seven skin metabolites in response to capsaicin supplementation were shown. These metabolites were mainly associated with energy metabolism, lipid metabolism, and oxidative stress. Using Kyoto encyclopedia of genes and genomes pathway enrichment analysis, it was shown that these altered metabolites were involved in some important pathways, such as central carbon metabolism in cancer, pyruvate metabolism, ABC transporters in plasma as well as sphingolipid metabolism in the skin (Xiao et al., 2022).

The exact role of TRPV1 in metabolic disorder associated with lipid metabolism, however, is still debatable. Important issues regarding its activation and desensitization mechanism in response to many agonists are still unclear. For example, it was shown that activation or upregulation of this receptor may help to treat or mitigate metabolic disorder (see review by Wang et al., 2011; Zhang C. et al., 2020). In contrast, it was shown that inhibition or downregulation of this receptor may provide a beneficial therapeutic option for metabolic disorder (Motter and Ahern, 2008; Marshall et al., 2013; Harb et al., 2019; Singh et al., 2020; Cocci et al., 2021; Wu et al., 2021). These two contradictory points of view will receive a special focus in this review in addition to shedding light on the role of Ca^2+^ in hyperlipidemia, the potential molecular mechanisms underlying TRPV1 regulation in lipid metabolism, and lipid mediators for TRPV1. In order to understand the role of lipids and drugs in TRPV1 regulation, we will briefly examine the structure of TRPV1.

## 2 Role of calcium in lipid metabolism

In resting cells, the cytoplasmic [Ca^2+^] is kept at low levels where Ca^2+^ homeostasis is maintained through Ca^2+^ transporters, sensors and buffering proteins distributed throughout the plasma membrane, cytoplasm, nucleus, mitochondria, and endoplasmic reticulum (ER). Increased intracellular [Ca^2+^] can lead to cellular damage where prolonged disruption of Ca^2+^ homeostasis often contributes to various chronic pathological developments (Chen et al., 2022). Among the many metabolic pathways normally affected by a change in [Ca^2+^] is lipid metabolism in hepatocytes and other cell types. Lipid accumulation is caused and/or exacerbated by altered intracellular Ca^2+^ homoeostasis through several mechanisms such as decreased lipolysis, inhibition of lipid autophagy, inhibition of beta-oxidation, increased lipogenesis and decreased secretion of lipids to the blood (Ali et al., 2019). For instance, chronic elevation of [Ca^2+^]_i_ in hepatocytes during obesity and lipotoxicity was found to inhibit autophagic flux by preventing the fusion of autophagosomes and lysosomes. This effect was restored using the calcium channel blocker verapamil which caused increased autophagosome-lysosome fusion in liver, prevented accumulation of protein inclusions and lipid droplets and suppressed inflammation and insulin resistance (Park et al., 2014).

**FIGURE 1:**
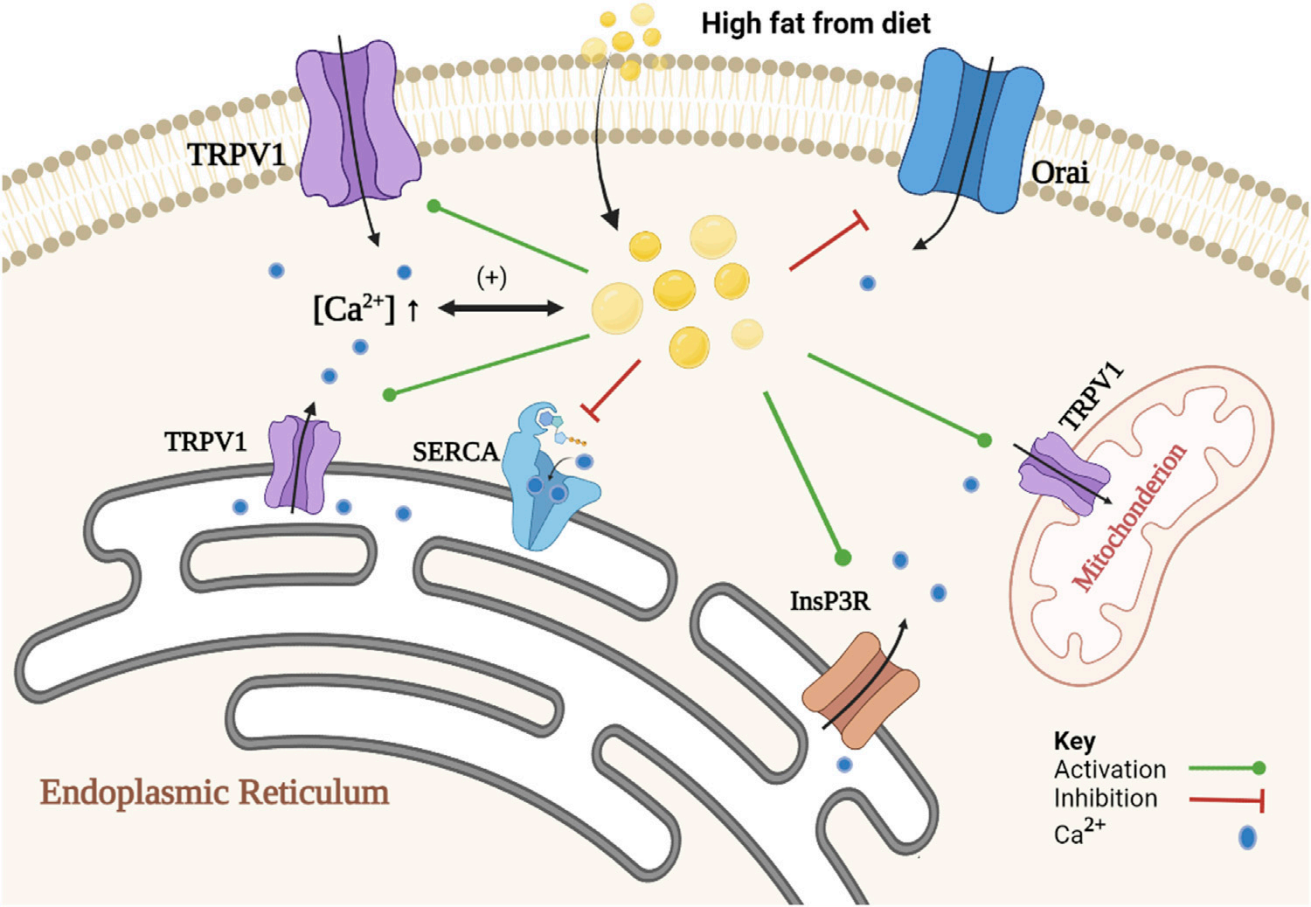
Schematic representation of the association of Ca^2+^ and lipid accumulation in steatotic hepatocytes. The proposed mechanism is a positive feedback loop between alteration in Ca^2+^ homeostasis and formation of cytoplasmic lipid droplet. Alteration in Ca^2+^ homeostasis promotes lipid synthesis and inhibits lipid degradation. An increase in [Ca^2+^]i inhibits autophagy of cytoplasmic lipid droplets, and a decrease in SOCE activity causes inhibition of lipolysis, and an increase in [Ca^2+^]_MT_ inhibits beta-oxidation pathway. In contrast, lipids induce changes in Ca^2+^ homeostasis through inhibition of SOCE (e.g., Orai) and SERCA2b activities. Lipids also activate InsP3R indirectly and increase the expression of TRPV1.

On the other hand, numerous studies showed that lipids induce alteration in intracellular Ca^2+^ signaling in steatotic hepatocytes (fatty liver), allowing further lipid accumulation. Elevation in the concentrations of certain lipids such as un-esterified cholesterol, saturated free fatty acids, diacylglycerols, lysophosphatidyl-choline, sphingolipids, ceramide, leukotrienes and/or prostaglandins in non-adipose tissue leads to cellular dysfunction and to lipotoxicity (Ali et al., 2019). Such lipids disrupt intracellular Ca^2+^ homeostasis by increasing free [Ca^2+^] in the cytoplasm and mitochondrial matrix, and decreasing [Ca^2+^] in the ER. Ca^2+^ channels and transporters, that are being affected in steatotic hepatocytes, are the sarco/endoplasmic reticulum Ca^2+^ATPase2b (SERCA2b), type 1 inositol trisphosphate receptor (InsP3R1), store-operated Ca^2+^ entry (SOCE) channels (Ali et al., 2019; Chen et al., 2022) and TRPV1 (Zhao and Tsang, 2017; Harb et al., 2019). It is important here to note that the evidence for the presence of functional TRPV1 on the plasma membrane of primary hepatocytes is limited. Most of evidence was obtained from HepG2 cells, or some other cell lines, which may not represent the physiological role of TRPV1 in normal hepatocytes. For example, Eberhardt et al. (2017) failed to observe any increase in intracellular calcium when capsaicin was applied in increasing concentrations (1–100 μM) using calcium imaging of Fura-2-AM loaded primary cultured mice hepatocyte. Further studies are needed to emphasize the localization of TRPV1 in normal liver cell and its role in the normal physiology of the liver. Figure 1 represents the role of Ca^2+^ in lipid metabolism and the effect of lipid on Ca^2+^ signaling.

## 3 General structure of TRPV1

**FIGURE 2:**
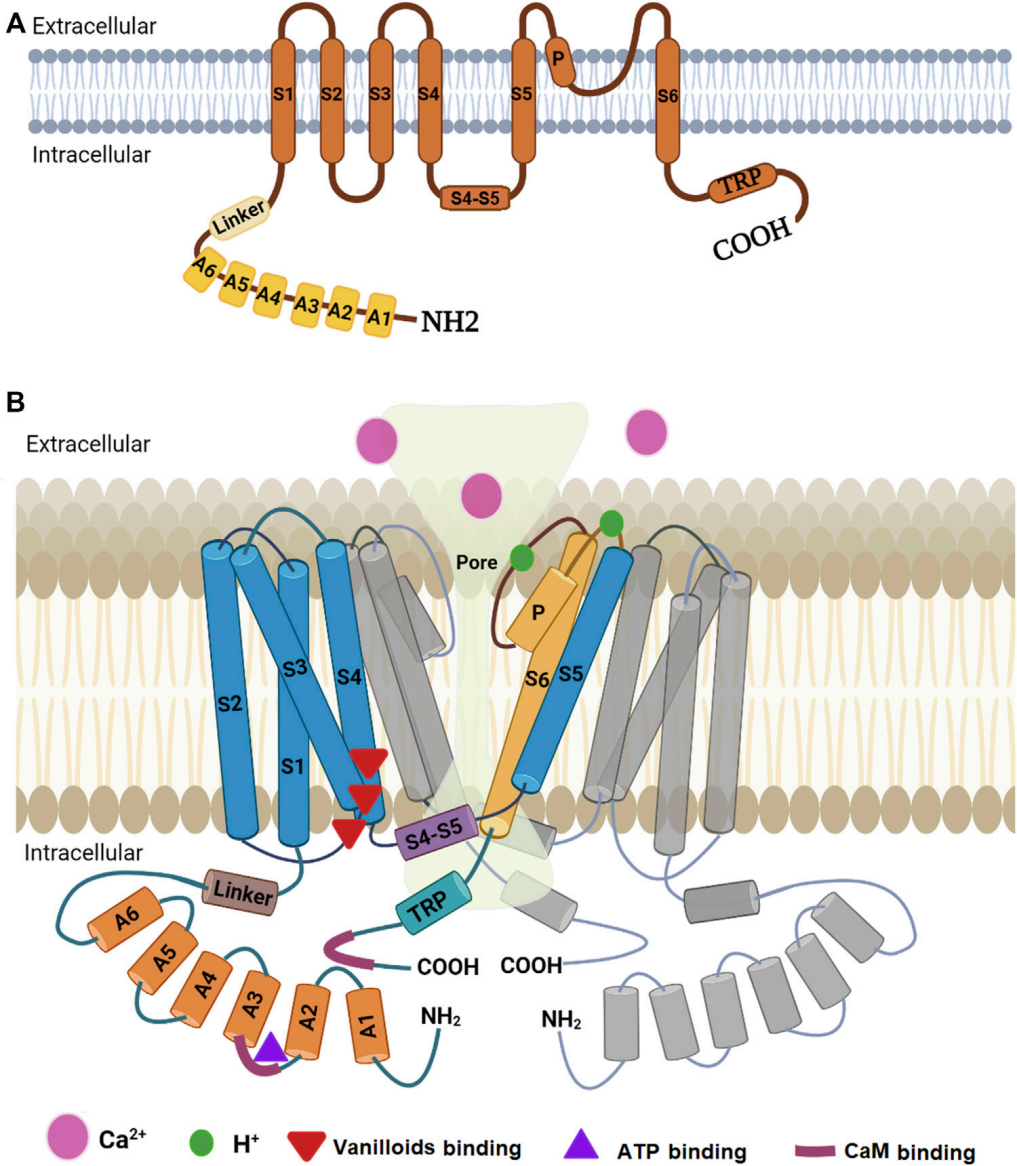
Diagrammatic structure of TRPV1. **(A)** One subunit showing N-terminus containing six ankyrin subunits (A1-A6) and a linker, transmembrane region with six helical segments (S1-S6), a pore loop (P), and a C-terminus containing a TRP domain. **(B)** Two monomers of the TRPV1 with a pore in the middle. One monomer (colored) is closer to the reader while the other (grey colored) is at 180°. The other two monomers have been omitted to show the pore. Part B is modified from the work of Shuba (2021).

Determination of the high-resolution structure of TRPV1 was not easy due to the experimental difficulties in crystallizing this type of protein. Lately, our knowledge on the structure of a mammalian TRPV1 was greatly enhanced by obtaining a high-resolution structure through single-particle cryo-electron microscopy (Liao et al., 2013). This technique facilitated breaking the side-chain resolution barrier for membrane proteins without crystallization. Using this analysis, Liao et al. (2013) were able to show that TRPV1 exhibits four-fold symmetry around a central ion pathway formed by transmembrane segments 5-6 (S5-S6) and the intervening pore loop, which is flanked by S1-S4 voltage-sensor-like domains (Figure 2). TRPV1 has a wide extracellular ‘mouth’ with a short selectivity filter. The conserved TRP domain, a 23-25-amino-acid-long region located just after S6, interacts with the cytoplasmic S4-S5 linker and contributes to allosteric modulation of channel gating. Subunit organization is facilitated by interactions among cytoplasmic domains, including the amino-terminal ankyrin repeats (Liao et al., 2013). Furthermore, it was found that rodent TRPV1 protein has a long N-terminus that contains multiple ankyrin repeats, and a relatively short C-terminal region. These intracellular domains have crucial role in the interactions with other proteins and they contain binding sites for molecules that regulate TRPV1 function (i.e., calmodulin or CaM and ATP binding sites located at the N- and C- termini) (Benítez-Angeles et al., 2020). Ankyrin repeats were isolated and crystallized and their structure was determined by X-ray diffraction. This technique revealed the structure of six ankyrin repeats of TRPV1, each composed of a typical 33-amino acid motif forming antiparallel α-helices followed by a finger loop. These motifs generate surfaces available for interactions with ankyrins from other proteins. Moreover, the TRPV1 ankyrin repeats showed an electron density corresponding to an ATP molecule bound to these structures, which has been shown to positively regulate TRPV1 activation (Benítez-Angeles et al., 2020). In human homologue, the crystal structure of a ligand-free form of TRPV1 ankyrin repeat domain showed a unique conformation in finger loop 3 near Cys258, which is most likely to be involved in inter-subunit disulfide-bond formation. Also, this structural feature may be attributed to the high sensitivity of human TRPV1 to oxidants (Tanaka et al., 2020).

Sites in transmembrane domain S6 are important for gating by agonists and antagonists. TRPV1 is gated by vanilloids and endogenous inflammatory fatty acid metabolites that bind to sites close to the intracellular face of the membrane (Bessac and Jordt, 2008). The capsaicin pocket, in the human TRPV1 sequence, is a hydrophobic cavity formed by residues Y512, S513, T551, and E571 (Benítez-Angeles et al., 2020). In contrast, the binding sites for protons are conserved between species and located at the extracellular end of S5 (E600 and E648) and within the channel pore loop (Bessac and Jordt, 2008; Benítez-Angeles et al., 2020). Interestingly, protons bind to E600 amino acid residue and enhance TRPV1 activation by other stimuli such as temperature or capsaicin, whereas E648 specifically regulates activation by protons (Benítez-Angeles et al., 2020).

The C-terminal domain plays a critical role in determining and fine-tuning of the channel response to agonists. Presence of positively charged domains within the C terminus of TRPV1 may function as interaction site for the substrate of phospholipase C, phosphatidylinositol-4,5-bisphosphate (PIP2), to exert an inhibitory effect on channel function (Bessac, and Jordt, 2008). This conclusion was substantiated by Caires et al. (2021) who found that TRPV1 C-terminal domain is required for the phosphoinositide lipid-mediated inhibition of the channel, and the aversive response to capsaicin in the worm *Caenorhabditis elegans*. Actually, it was found that deletion of the distal C-terminal domain (Δ764) of TRPV1 prevents the interaction with phosphoinositides, allowing capsaicin to evoke more Ca^2+^ *in vivo* and enhancing the aversive response of the worm to capsaicin independent of the phosphoinositide lipid content.

Based on ligand-bound TRPV1 structures, it was found that there are different pore profiles in this channel which implies that each agonist can lead to a different gating mechanism (Benítez-Angeles et al., 2020). Structure-function studies predicted the vanilloid binding domain to be located in transmembrane domains S3-S4 and S5-S6 (Johnson et al., 2006; Bessac and Jordt, 2008). Several studies, however, revealed that TRPV1 has many modes of activation resulting from different binding sites for several TRPV1-activating compounds. For example, the endocannabinoid anandamide mode of binding was favored between S1-S4 region in the channel but also with lower probability of binding to the vanilloid binding pocket (Muller et al., 2020).

## 4 Modulation of TRPV1 by lipids

In Section 5 below we will discuss the evidence that TRPV1 regulates lipid metabolism in a complicated manner, but it is interesting to note that lipids also modulate the expression and the functional activity of TRPV1. Various types of lipids such as cholesterol, phosphoinositide, estrogen, oxytocin, oleoylethanolamide affect the activity and/or the expression of TRPV1 (Almási et al., 2008; Picazo-Juarez et al., 2011; Pohóczky et al., 2016; Bowen et al., 2017; Caires et al., 2021).

The contribution of TRPV1 channels in lipid metabolism has been previously reviewed but many questions about the mechanism of action and the interaction of these channels and lipids remain unresolved. Here, we are trying to updates our understanding of the role of TRPV1 in cholesterol and lipid metabolism and provides an in-depth discussion of the complexity of their mechanism of actions.

### 4.1 Lipid-dependent gating of TRPV1

Although stimulation of TRPV1 affects lipid metabolism, lipids are also one of the main regulators of the activity of TRPV1. Different types of lipids such as phospholipids, fats, and steroids influence the functional activity of TRPV1. Endogenous cannabinoids such as anandamide and over 15 additional endogenous lipids enhance the function of TRPV1 either as allosteric effectors or as direct agonists, or both (Zhang C. et al., 2020; Zhai et al., 2020; Manchanda et al., 2021). Some lipids like endovanilloids increase the probability of TRPV1 channel gating indirectly by phosphorylation (De Petrocellis and Di Marzo, 2005).

Annular lipids have been hypothesized to mediate the allosteric coupling between the vanilloid site and the peripheral cavities (PCs) leading ultimately to TRPV1 activation. Activation of TRPV1 requires the rotation of a conserved residue on the S6 segment from the S4-S5 linker towards the pore. This movement is associated with dehydration of PCs located between S6 and the S4-S5 linker, and with hydration of the pore. Using the multi-microseconds molecular dynamics simulations and free energy calculations of several TRPV1 systems revealed that annular lipids have a peculiar “buried” conformation upon concomitant binding of vanilloids and spider venom toxin. It is worth noting that lipids in this conformation project the hydrophobic tails into the PCs, thereby enhancing their dehydration and the consequent opening of the channel (Gianti et al., 2019).

**FIGURE 3:**
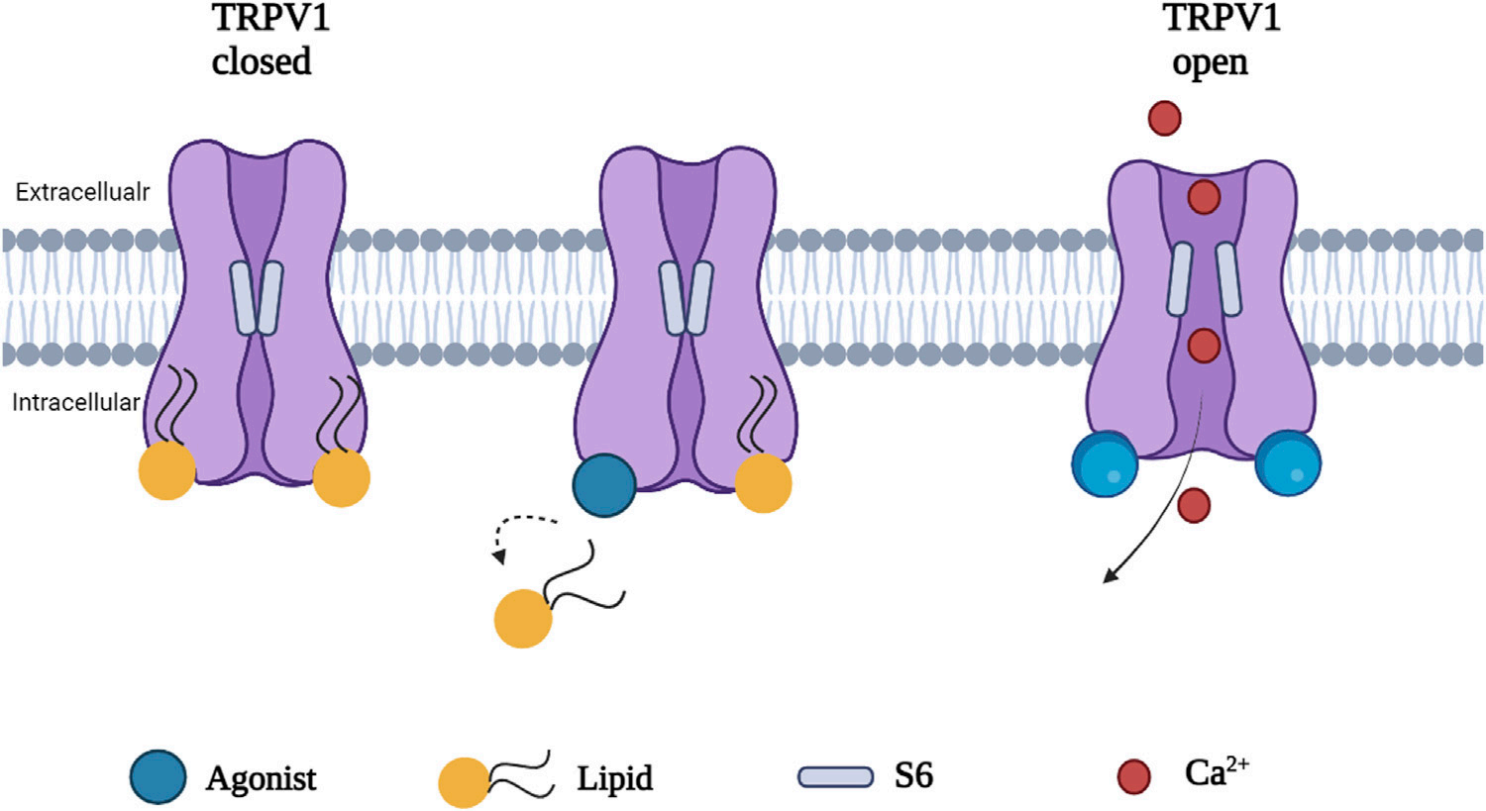
A proposed mechanism of TRPV1 activation. Agonist induced conformational change in TRPV1 by full displacement of the regulatory lipids in all subunits enhances sufficient S5 movement, allowing S6 movement and opening the channel.

Phosphatidylinositol (PI), a silent resident lipid, has been suggested to serve an essential role in allosteric activation of TRPV1 by vanilloid compounds and analogs. Both PI and capsaicin or resiniferatoxin have been shown to share the same inter-subunit binding pocket between a voltage sensor-like domain and a pore domain in TRPV1. The binding of capsaicin in the vanilloid pocket exhibited a sequential cooperativity mechanism which is lipid-dependent. *In silico* research suggested that anchor-stereoselective sequential cooperativity between an initial recessive transient silent weak ligand binding site and a subsequent dominant steady-state strong ligand binding site in the vanilloid pocket may facilitate the release of the occluded resident lipid (i.e. PI) for allosteric activation of TRPV1 by vanilloids or analogs through non-covalent interactions (Wang, 2021). A recent study reported the use of cryo-electron microscopy to visualize conformational changes of TRPV1 in response to different stimuli such as protons, vanilloid agonists, and peptide toxins. A competition between the resident regulatory PI lipid and vanilloid agonists within the vanilloid binding pocket (VBP) in a two-step process has been observed. Vanilloid agonists displace the regulatory PI lipid, thereby competing off the aliphatic tail of PI followed by displacement of the inositol head group. Notably, endogenous PI lipids must be fully displaced by the agonist in all subunits to induce sufficient S5 movement allowing S6 movement which is necessary to open the lower gate (Figure 3) (Zhang et al., 2021).

Melnick and Kaviany (2018) emphasized the role of embedded lipids in the thermally induced transition of TRPV1 from its closed to open configuration using all-atom molecular dynamics simulations of its most recently resolved structure at different temperatures. They proposed the heat activation mechanism of TRPV1. Heat causes destabilization in a hydrophobic cluster in the C-terminal (CT) domain, allowing a new cluster to form between the key residues (R739, R743) in CT and the adjacent one-turn helix of the linker domains. This pulls the linker domain away from the VBP, breaking the hydrogen bonds between the linker and S2-S3 link, and opening the VBP. Afterwards, the lipid in the VBP is displaced or completely ejected and a new hydrogen bond forms between S4 and S5, pulling S6 away from the ion channel and opening the gate (Melnick and Kaviany, 2018). An excellent cartoon of this mechanism of lipid modulation of TRPV1 gating is found in Melnick and Kaviany (2018).

Phosphoinositides are phosphorylated derivatives of PI which consist of seven species generated by reversible phosphorylation of the inositol moieties at the positions 3, 4, and 5 and have significant physiological roles (Nakada-Tsukui et al., 2019). In particular, PIP2 regulates several ion channels including members of TRP family. Phosphoinositides modulate TRPV1 activity in a complex and controversial manner and have both positive and negative effects. Phosphatidylinositides have a dual role through their interactions at the single site within the TRPV1 C-terminus, and they may function as negative allosteric modulators by acting as endogenous, tightly bound co-factors that stabilize TRPV1 in its resting state and, therefore, serve as competitive vanilloid antagonists. Alternatively, phosphatidylinositides may function as positive, obligatory co-factors that bind to TRPV1 in the closed state to sensitize the channel for subsequent activation by vanilloids or other stimuli (Gao et al., 2016).

Recently, phosphoinositides have been found to regulate channel activity *via* functionally distinct binding sites, which may help explaining some of the complexities of the effects of these lipids on TRPV1. It has been shown that PI, but not PI4P, partially inhibited channel activity in the presence of PIP2 in excised inside out patches. The inhibitory effect showed an inverse correlation with the concentration of capsaicin. In addition, mutating residues that are predicted to interact with PI, but not with capsaicin, caused higher sensitivity to capsaicin activation and reduced inhibition by PI. These data are compatible with the concept that PI is acting as a competitive vanilloid antagonist. On the other hand, in the absence of PIP2, PI partially stimulated TRPV1 activity especially in the presence of high capsaicin concentrations, an indication that PI also binds to an activating lipid binding site. The molecular modeling was used to characterize the binding of phosphoinositides to the inhibitory site which partially overlaps with the vanilloid binding site and the stimulatory site located between the S4-S5 linker and the proximal C-terminal TRP-domain. Theoretical structural models are consistent with the hypothesis that phosphoinositides act primarily on these non-overlapping binding sites, one with activating and the other with inhibiting effect (Yazici et al., 2021). Furthermore, not only the site of interaction of phosphoinositides on TRPV1 channel shapes the outcome, but the amount of phosphoinositides is also important since it has been shown that the activity of TRPV1 is enhanced when the phosphoinositide lipid content is reduced (Caires et al., 2021).

Cholesterol in membrane is another important regulator of TRPV1 activity. It is a major component of animal cell plasma membranes where it acts as buffer to maintain membrane fluidity. Importantly, cholesterol modifies the function of many classes of ion channels indirectly through altering the thickness and the elastic properties of the surrounding lipid bilayer. In addition, the function of many protein channels is altered by specific interactions between cholesterol and these protein channels. Many experiments indicated the presence of a cholesterol-binding site in the TRPV1 S5 helix that prevents channel opening when occupied by cholesterol (Picazo-Juarez et al., 2011). As expected, cholesterol binding motifs were found in the transmembrane domains of TRPV1 channels because cholesterol is an integral membrane lipid and should interact with the transmembrane domains of these channels (Barbera and Levitan, 2019). Interestingly, it was found that mammalian TRPV1 currents were not changed by low cholesterol concentration, but were markedly decreased by high cholesterol concentration, indicating that TRPV1 function was modulated by cholesterol concentration in the plasma membrane (Picazo-Juarez et al., 2011). Table 1 shows the binding sites of different lipids and their regulatory role on TRPV1 using different research methods.

**TABLE 1:** Types of lipids affecting the gating of TRPV1 with their binding sites and observed effects.

Lipid	Binding site in TRPV1	Type of research	Effect on TRPV1	Reference
Annular lipids	Between the vanilloid site and PC	*In silico*	Mediate allosteric TRPV1 activation	Gianti et al. (2019)
Phosphatidylinositol	Vanilloid binding pocket	*In silico*; Cryo-electron microscopy	Allosteric activation of TRPV1 by vanilloid compounds and analogs	Wang, (2021), Zhang et al. (2021)
Phosphatidylinositides	C-terminus	Cryo-electron microscopy with lipid nanodisc technology	Negative allosteric modulators (act as competitive vanilloid antagonist)	Gao et al. (2016)
			Positive allosteric modulators (act as obligatory co-factors)	
Phosphoinositides	Vanilloid binding site (inhibitory site); Between the S4-S5 linker and proximal C-terminal TRP-domain (activating site)	Cryo-electron microscope	Dual regulation; inhibiting and activating effects by occupying different binding sites	Yazici et al. (2021)
		*In vivo*, using transgenic TRPV1 worms (*C. elegans*)	Phosphoinositide content decreased, the TRPV1 activity increased	Caires, et al. (2021)
Cholesterol	S5 helix	Capsaicin-activated currents in excised patches from TRPV1-expressing HEK cells; Site-directed mutagenesis	Prevents channel opening; When cholesterol concentration increased TRPV1 activity decreased	Picazo-Jua´rez et al. (2011)

### 4.2 Lipid affects the expression and functional activity of TRPV1

Lipid mediators, such as ox-LDL, have been reported to increase the activity of TRPV1 in bone marrow-derived macrophages, allowing increased [Ca^2+^]_i_ level, and inducing foam cell formation (Zhao et al., 2013). Also, the role of the endogenously produced omega-3 lipids, like 17,18-epoxyeicosatetraenoic acid (17,18-EEQ), in modulating the activity of sensory neurons was investigated and it was found that 17,18-EEQ uses prostacyclin receptor and sensitizes the TRPV1 and TRPA1 receptors (Schäfer et al., 2020). Indeed, epoxidation of N-arachidonoyl-dopamine (NADA) and N-arachidonoyl-serotonin (NA5HT) by epoxygenases generates epoNADA and epoNA5HT. These epoxide metabolites are bioactive lipids that serve as modulators of the endocannabinoid-TRPV1 axis. EpoNADA and epoNA5HT are more potent modulators of TRPV1 than their parent molecules, NADA or NA5HT. It was found that NADA and epoNADA are full agonists of TRPV1 in TRPV1-transfected HEK cells. EpoNA5HT, in particular, displays a strong antagonistic action of TRPV1 expressed in either HEK293 cells or native DRG neurons and an effective agonist of CB1 (Arnold et al., 2021). The steroid hormone estrogen induced up-regulation of TRPV1 in the endometrium of immature rats (Pohóczky et al., 2016). Furthermore, the expression of TRPV1 in bladder arterioles is found to be sex-dependent. It was demonstrated that the expression of TRPV1 in bladder arterial and arteriolar smooth muscle in post-pubertal female mice was increased in comparison with that of males. These observations may be correlated with the role of sex hormones, estrogen in particular (Phan et al., 2016).

Oleoylethanolamide (OEA) is an endogenous lipid mediator that stimulates fatty acid uptake, lipolysis, and *β*-Oxidation, and reduce food intake (Bowen et al., 2017). Acting as a TRPV1 receptor antagonist or weak partial agonist both *in vitro* and *in vivo* (Almási et al., 2008), OEA may serve as a link between enterocytes and the central nervous system to induce satiety. OEA activates TRPV1 and AMPK, thus causing downregulation of TLR4/NF-κB pathway and therefore exhibiting anti-inflammatory effects (Yao et al., 2019). Moreover, one of the most endogenous lipid activator of TRPV1 is 20-hydroxyeicosatetraenoic acid (20-HETE) which protects the heart against cardiac dysfunction induced by LPS. This effect, in part, underlies the release of calcitonin gene related peptide in endotoxemia. Interestingly, inhibition of lipid biosynthesis decreases TRPV1-dependent cardioprotective effects in WT mice caused by low-dose LPS (Chen et al., 2018). Recently, 20-HETE, produced from membrane lipids by stimuli like LPS, was found to activate TRPV1 on C-fiber, causing their activation and release of substance P to cause skin edema. 20-HETE was also detected locally at the site of an inflammatory response in cantharidin-induced skin blister in human. Therefore, it has been suggested that blocking the 20-HETE-TRPV1 pathway may provide useful therapeutics for inflammatory skin disorders (Hamers et al., 2022). Recently, lipid nanoparticles were used successfully for capsaicin encapsulation to optimize capsaicin release, reduce pain and avoid TRPV1 internalization and degradation caused by a prolonged activation of this receptor (Puglia et al., 2020). Table 2 shows the modulatory effect of lipids on TRPV1 using various experimental techniques.

**TABLE 2:** Types of lipids affecting the expression and/or the activity of TRPV1.

Lipid	Type of research	Effect on TRPV1	Reference
ox-LDL	*In vitro*, measurement of [Ca^2+^]𝑖 after incubation with oxLDL in BMDMs pretreated or not with capsazepine	TRPV1 activity increased	Zhao et al. (2013)
17,18-EEQ	*In vitro,* calcium imaging of murine dorsal root ganglion cells stimulated twice with capsaicin and with 17,18-EEQ	Activation of TRPV1	Schäfer et al. (2020)
NADA, NA5HT, epoNADA and epoNA5HT	*In vitro*, activation of TRPV1 was determined using Fura 2-AM Ca^2+^-influx assay in TRPV1-transfected HEK cells; *In vitro,* ratiometric Ca^2+^ imaging of cultured wild-type mouse DRG neurons	NADA and epoNADA increased TRPV1 activity; NA5HT and epoNA5HT decreased TRPV1 activity	Arnold et al. (2021)
Estrogen	*In vivo*, RT-PCR was used to determine gene expression of TRPV1 in the endometrium of female Wistar rats treated with estrogen. Semi-quantitative scoring of TRPV1 immunopositivity was performed in the rat endometrium	TRPV1 up-regulated and the activity increased	Pohóczky et al. (2016)
	*In vitro*, [Ca^2+]^i was measured in primary culture of rat endometrial cells pre-incubated with estrogen	Expression of functional TRPV1 in bladder arteriolar smooth muscle in post-pubertal female mice increased	Phan et al. (2016)
	*In vivo*, fluorescence imaging, PCR and quantitative PCR were used to determine the expression of TRPV1 in the bladder of male and female mice		
	*In vitro*, Ca^2+^ imaging of isolated arterioles loaded with Fluo-4		
OEA	*In vitro*, Western blot was used for determination of protein expressions of TRPV1/AMPK, and calcium imaging for measuring [Ca^2+]^i in bone marrow-derived dendritic cells treated with OEA	TRPV1 activation	Yao et al. (2019)
20-HETE	*In vivo*, cardiac function, body temperature, and heart rate were measured in WT (TRPV1^+/+^) C57BL/6 mice treated with LPS and the 20-HETE blocker, 17-octadecynoic acid	TRPV1 activation	Chen et al. (2018)
	*In vitro*, calcium fluorescence was used to measure the activity of TRPV1 in HEK cells transfected with hTRPV1 and incubated with the 20-HETE blocker, 17-octadecynoic acid, or the TRPV1-specific blocker AMG-9810	TRPV1 activation	Hamers, et al. (2022)
	*In vivo*, human cantharidin-induced blister samples and inflammatory responses in TRPV1 transgenic mice		
Capsaicin encapsulated by lipid nanoparticles (Cps-LNP)	*In vivo*, pain was induced in male CD1 mice by subcutaneous injection of capsaicin or Cap-LNP. Licking, lifting, and shaking behavior of the injected paw were measured. The expression of TRPV1 in the skin of male CD1 mice (at the site of injection) was evaluated using Western blot analysis	The expression and the activity of TRPV1 increased	Puglia et al. (2020)

## 5 Role of TRPV1 in metabolic syndrome associated with hyperlipidemia

Metabolic syndrome is a combination of multiple metabolic disorders that increase the incidence of cardiovascular diseases (Bratz et al., 2008). It includes hypertension, insulin resistance, obesity and hyperlipidimia, which are more or less related to dysregulation of lipid metabolism (Hu et al., 2013). Molecular mechanisms underlying lipid regulation are still unclear, however, TRPV1 has been suggested to be involved in the process, and despite considerable research efforts to understand the role of TRPV1 in the metabolic syndrome, the mechanism of action remains unresolved. This is because TRPV1 has many binding sites other than vanilloid binding pocket (VBP) that can be occupied by ligands, and there are different modes of activations, therefore producing different effects (Muller et al., 2020).

### 5.1 TRPV1 expression is enhanced in metabolic syndrome

A mounting evidence demonstrates that TRPV1 knockout mice are resistant to obesity induced by HFD (Cocci et al., 2021), suggesting that TRPV1 has a role in maintaing lipid homeostasis (Zhao et al., 2013). Cumulative experimental evidence showed that expression and/or activity of TRPV1 is increased in metabolic syndromes, such as in atherosclerosis (Zhao et al., 2013; Zhao et al., 2016), obesity (Motter and Ahern, 2008; Marshall et al., 2013; Singh et al., 2020; Cocci et al., 2021), hyperlipidemia and NAFLD (Wu et al., 2021), indicating that TRPV1 is involved in the signaling pathway that favors fat accumulation. Thus, shutting down of this signaling pathway with TRPV1 antagonists or agonists that cause desensitization may counteract fat accumulation (Motter and Ahern, 2008). Zhao et al. (2016) emphasized the link of TRPV1 channels to Ca^2+^-dependent calpain activity in excess NO-deregulated cholesterol metabolism. They concluded that ox-LDL or pro-inflammatory cytokines induced lipid accumulation and inflammation in macrophage-foam cells, thereby inducing iNOS expression and excess NO production. Nitric oxide induced TRPV1-Ca^2+^-calpain signaling that leads to downregulation of protein expression of liver X receptor α (LXR) and ATP binding cassette subfamily A1 (ABCA1). Furthermore, oxLDL-induced lipid accumulation in macrophages was ameliorated by TRPV1 agonists but exacerbated by TRPV1 antagonits. The protective effects of TRPV1 agonists may be due to the activation of LXRa since inhibition of LXR activation by siRNA diminished the TRPV1-agonist-mediated upregulation of ABCA1 and ABCG1. Treatment with oxLDL increased TRPV1 channel activity in bone-marrow-derived macrophages (BMDMs), as evidenced by a TRPV1-mediated increase in [Ca^2+^]*i* level to a profile similar to that evoked by TRPV1 agonists. Therefore, activation of TRPV1/Ca^2+^ signaling may inhibit the formation of foam cells *in vitro* since removal of Ca^2+^ by EGTA aggravated the ox-LDL lipid accumulation in BMDMs (Zhao et al., 2013). This signaling pathway impairs reverse cholesterol efflux and causes pathologic accumulation of lipids in macrophages (Zhao et al., 2016). Consistently, proteomic and transcriptomic analyses revealed that activation of TRPV1 by cannabinoid caused upregulation of cholesterol biosynthesis machinery in human keratinocyte and neuroblastoma cell lines. Such effect is mediated by an increase in cytosolic calcium, and activation of AMPK and ERK kinases (Guard et al., 2020). A recent study showed that activation of TRPV1 by capsaicin plays a modulatory role in the biosynthesis and metabolism of a wider range of lipid signaling molecules in hTRPV1-HEK cells. Lipidomics analysis revealed that the level of 2-acyl glycerols, including 2-arachidonoyl-sn-glycerol, was increased and the level of N-acyl ethanolamine, including N-arachidonoyl ethanolamine, was decreased by capsaicin in a time, dose and temperature-dependent manner (Manchanda et al., 2021).

Therefore, a decrease in the expression of TRPV1 has been suggested to protect against metabolic syndrome. To prove this aspect, gene knockout was used to explore the importance of TRPV1 by comparing the TRPV1 knockout organism to a wild type with a similar genetic background. It was found that knockout of TRPV1 protected against diet-induced obesity (Motter and Ahern, 2008). Motter and Ahern (2008) showed that TRPV1-null mice gained less body mass than WT mice fed HFD. For adiposity, TRPV1-null mice had lower abdominal and subcutaneous fats, smaller adipocytes, lower hepatic fat droplets than their WT counterparts. They proposed that the molecular mechanism by which TRPV1 influences energy and lipid metabolism is through insulin signaling. They suggested that activation of TRPV1 in sensory nerve terminals in mouse 3T3-L1 preadipocytes may trigger the secretion of two neuropeptides: substance P and calcitonin gene related peptide (CGRP) which are known to modulate pancreatic islets function. They found that 3T3-L1 preadipocytes exhibit functional responses to CGRP and express the CGRP receptor, CRLR. Importantly, increased level of CGRP is associated with insulin resistance and obesity (Motter and Ahern, 2008).

Deletion of TRPV1 also protected from obesity-induced hypertension, low-grade inflammation, and glucose tolerance (Marshall et al., 2013). Histological examination of a cross section of aorta revealed that vascular hypertrophy parameters such as the vascular wall width, collagen deposition, and total aortic wall width were significantly decreased in HFD-fed TRPV1 knockout mice in comparison with HFD-fed WT counterparts. Furthermore, plasma glucose levels were improved in the HFD-fed TRPV1 knockout mice compared to the HFD-fed WT mice. The inflammatory cytokine, interleukin 10 and interleukin 1β, decreased in HFD-fed TRPV1 knockout mice compared to the WT mice (Marshall et al., 2013).

To study this issue, pharmacological and nutritional manipulations along with different research techniques like qRT-PCR, Western blot and immunohistochemistry were used. Using immunohistochemistry, Harb et al. (2019) showed that the phenol-like eugenol attenuated NAFLD by down regulating hepatic TRPV1 (Harb et al., 2019). Likewise, suppression of hepatic TRPV1 expression by total sesquiterpene glycosides from loquat leaves protected from HFD-induced NAFLD (Wu et al., 2021). Using olanzapine to increase food intake, weight gain, and adiposity index through modulation of hypothalamic appetite control, Singh et al. (2020) reported increased expression of TRPV1/TRPV3 whereas berberine attenuated the effect of olanzapine-induced metabolic changes by modulating TRPV1/TRPV3 (Singh et al., 2020). Cocci et al. (2021) found that the bioactive ingredients of tart cherry seed and juice attenuated lipogenesis in visceral adipose tissue by modulating the interplay between cannabinoid receptor (CB1), PPARr and TRPV channel gene transcription (Cocci et al., 2021). Similarly, using AMG517 as a specific antagonist to block TRPV1 receptors enhanced activity-dependent energy in mice as evidenced by increased oxygen consumption, fat oxidation, and locomotor activity (Hai et al., 2020) and this effect disappeared when sensory nerve ending to the stomach were desensitized by capsaicin.

Interestingly, diet-induced metabolic disorders are also alleviated by chronic use of TRPV1 agonists such as capsaicin. Capsaicin reduced lipid deposition in atherosclerotic lesions induced by ox-LDL (Zhao et al., 2013). Although ox-LDL and vanilloids like capsaicin are agonists of TRPV1, they activate TRPV1 in different modes and subsequently they have different mechanism of action. Ox-LDL induces iNOS expression, increases NO production, activation of TRPV1, and Ca^2+^ influx, which in turn increases calpain activity, promotes LXRα degradation and downregulates protein expression of LXRα and ABCA1, to subsequently impair reverse cholesterol efflux (Zhao et al., 2016), whereas vanilloids and other TRPV1 agonists regulate lipid metabolism by affecting various targeting molecules as described in Section 6 below.

Desensitization of TRPV1 is thought to be the mechanism explaining the paradoxical effectiveness of the TRPV1 agonists. Sanz-Salvador et al. (2012) have shown that prolonged exposure of TRPV1 to agonists like capsaicin decreased the total amount of receptors in a dose- and time-dependent manner by receptor endocytosis and downregulation through lysosomal degradation, resulting in a loss of function known as nociceptor defunctionalization (Sanz-Salvador et al., 2012; Puglia et al., 2020). This endocytotic route was clathrin- and dynamin-independent internalization, triggered by TRPV1 activation and Ca^2+^ influx, and inhibited by protein kinase-dependent phosphorylation of serine 116 (Sanz-Salvador et al., 2012). In consistence with these findings, we also demonstrated that chronic consumption of eugenol protected against hypercholesterolemia and fatty liver disease by causing an initial activation as an agonist, followed by long-term desensitization of TRPV1, suggesting that the pharmacological effect of eugenol may be mediated by downregulation of TRPV1 (Harb et al., 2019). In a similar manner, capsaicin and other vanilloids are used in pain relief since they selectively activate and consequently desensitize the nociceptive neurons. Activation of TRPV1 by vanilloids causes an elevation in intracellular free Ca^2+^ levels and triggers desensitization. Depending on the vanilloid concentration and duration of exposure, Ca^2+^ influx *via* TRPV1 desensitizes the channels themselves. This desensensitization may represent not only a negative feedback mechanism protecting the cell from toxic Ca^2+^ overload, but may also contribute to the analgesic effect induced by topical application of capsaicin (Vyklický et al., 2008). This desensitization strategy has been effectively used in various TRPV1 therapeutics, including capsaicin cream (Zostrix), and the high concentration patches (Qutenza). Additionally, oxytocin, an endogenous TRPV1 agonist, suppresses nociception and induces analgesia in animal models by specifically affecting inflammatory pain pathways, and such effect is mainly exerted by TRPV1 desensitization (Du et al., 2019).

It is important to note the difference in the effect between TRPV1-desensitising agonists and TRPV1 antagonists. Agonists can desensitize TRPV1 channels or even ablate TRPV1-expressing neurons whereas TRPV1 antagonists selectively inhibit TRPV1 activation. Therefore, the therapeutic effect as well as the side effect profiles for both classes of drugs are different (Deruyver et al., 2015).

### 5.2 TRPV1 expression is suppressed in metabolic syndrome

In the previous section, we discussed the studies which showed enhanced TRPV1 expression in metabolic syndrome. Some of these studies concluded that overexpression of TRPV1 is involved in the pathogenesis of metabolic diseases. In contrast, low expression of TRPV1 has been reported to be involved in metabolic syndrome (Baskaran et al., 2016; Krishnan et al., 2019; Wang et al., 2020). In apolipoprotein E-deficient mice, evodiamine significantly reduced atherosclerosis, hyperlipidemia, and systemic inflammation and this beneficially therapeutic effect was not seen in ApoE^−/−^ mice that lack TRPV1 (Wei et al., 2013), suggesting that TRPV1 curbs the development of atherosclerosis. A similar observation was shown by Connell et al. (2018) who showed that loss of TRPV1 gene in western-fed mice caused higher hepatic triglycerides, cholesterol and free fatty acid levels than wild type (WT) mice and this effect was more obvious in males than in females (Connell et al., 2018). Similarly, Baskaran et al. (2021) found that TRPV1 deficient mice developed metabolic dysfunction. They became obese, exhibited reduced locomotor activity, reduced energy expenditure, enhanced hepatic steatosis, and decreased thermogenic protein expression in adipose tissues. Bratz et al. (2008) conducted a study on porcine coronary arteries with metabolic syndrome induced by high cholesterol and fat diet and they measured TRPV1 channel mRNA and protein expression in coronary arteries from obese swine using real-time PCR and Western blot. Real-time PCR showed a significant increase in TRPV1 channel mRNA in coronary arteries from obese swine although Western blot analysis demonstrated a decrease in TRPV1 protein expression in the coronary arteries of obese swine. This discrepancy was attributed to transcriptional and posttranscriptional factors, including TRPV1 degradation, Ca^2+^ induced regulation of transcription factors, proteases, calpain, ubiquitin and proteasomes. Consistently, Li et al. (2013) showed that administration of capsaicin reversed free fatty acids-induced downregulation of TRPV1 expression in HepG2 cells and chronic dietary capsaicin prevented high fat diet (HFD)-induced reduction of TRPV1 expression in liver tissues of wild type mice (Li et al., 2013). Moreover, the expression of TRPV1 channel protein in the mammalian epididymal fat and subcutaneous fat pads from the inguinal region was suppressed by HFD in mice (Baskaran et al., 2016). A more recent study demonstrated the potential mechanisms of capsaicin in reducing dyslipidemia. Capsaicin seemed to increase the expression of TRPV1 originally suppressed by HFD in liver of Sprague Dawley rats, increased Ca^2+^ and this can activate AMPK pathway, thereby regulating glucose and lipid metabolism. In addition, capsaicin decreased the inflammatory factors IL-6 and IL-1β, improved the diversity and structure of gut microbiota, and affected the composition of bile acids (Gong et al., 2022).

Earlier, Li et al. (2012) showed that chronic consumption of capsaicin reduced lipid accumulation and triglyceride level in the liver from WT mice that have TRPV1 channel and fed HFD in comparison with mice that lack TRPV1 channel. They also tested the presence and the functional activity of TRPV1 channel in WT and TRPV1-null mice and found that TRPV1 mRNA and TRPV1 protein were expressed in hepatocyte from WT mice but not in hepatocyte from TRPV1-null mice. These authors also demonstrated that TRPV1 activation by dietary capsaicin enhanced hepatic PPARδ and autophagy-related proteins and reduced hepatic enzymes and inflammatory factor in WT but not in TRPV1^−/−^ mice (Li et al., 2013). They also showed using immunoblotting that TRPV1 long-time activation through chronic consumption of capsaicin upregulated hepatic uncoupling protein 2 (UCP2) in WT but not in TRPV1^−/−^ mice, suggesting that chronic activation of TRPV1 receptor might promote hepatic ß oxidation through upregulating UCP2.

TRPV1 protects the heart against cardiac dysfunction during endotoxemia which is used as a tool to induce or accelerate metabolic syndrome such as hyperlipidemia or obesity (Funk et al., 1993; Clemente-Postigo et al., 2019). The absence of TRPV1 promotes the development of cardiac dysfunction in mice as evidenced by reduced systolic contractility associated with reduced heart rate and body temperature after exposure to low-dose lipopolysaccharide (LPS). Low-dose LPS caused an increment in total TRPV1 protein and phosphorylated TRPV1 in heart tissue of WT mice in comparison with PBS-treated control mice. This increment was correlated with the activation of TRPV1 by the endogenously produced activator 20-HETE leading to the release of calcitonin gene related peptide (CGRP), which protects the heart against the cardiac dysfunction during endotoxemia (Chen et al., 2018). This study identified both TRPV1 and CGRP receptors as potential therapeutic targets in endotoxemia. Therefore, the presence of the active form of TRPV1 along with its endogenous activators in the heart indicates an important role of TRPV1 signaling pathway in preventing sepsis and maintaining cardiac function (Chen et al., 2018).

Table 3 summarizes the recent studies published on the role of TRPV1 in metabolic syndrome. Examining these studies shows that they approached metabolic syndrome by inducing different diseases (e.g., atherosclerosis, dyslipidemia, obesity, hypertension, diabetes, NAFLD) and they induced these diseases using different experimental protocols (e.g., high fat diet, chemicals like olanzapine, genetic manipulation) and different animal models (mice, rats, pigs) and cell lines (BMDM, HepG2, primary epididymal and subcutaneous preadipocytes) and measured the outcomes by different detection methods (e.g., qRT-PCR, Western blotting, immunohistochemistry, Ca imaging). This high degree of variability in the approach makes the extrapolation from one species/one protocol to another very difficult and impedes finding a unifying theme or making a solid conclusion. Therefore, further experiments are required to validate the contribution of TRPV1 in lipid regulation in both physiological and pathophysiological conditions.

**TABLE 3:** Expression of TRPV1 in various models of metabolic syndrome.

Metabolic syndrome	Model	Detection method	TRPV1 expression	Reference
Hyperglycemia, hyperlipidemia, and NAFLD	*In vivo*, HFD-induced insulin resistance in male C57BL/6 mice	Western blotting; Quantitative analyses	Hepatic TRPV1 protein increased	Wu et al. (2021)
Atherosclerosis	*In vivo*, ApoE^−/−^ mice; *In vitro*, BMDM exposed to ox-LDL	Western blotting; Quantitative analyses	TRPV1 protein increased in both models (atherosclerotic lesions of ApoE^−/−^ and BMDM)	Zhao et al. (2013)
Obesity	*In vivo*, HFD-induced obesity in male and female of WT and TRPV1-null mice	Calcium transients in adipocytes	3T3-L1 preadipocytes expressed functional CGRP, suggesting TRPV1-sensitive sensory nerves regulating energy and fat metabolism	Motter and Ahern, (2008)
Cardiovascular disease; hypertension, diabetes, and obesity	*In vivo*, diet-induced obesity in WT and TRPV1 knockout mice	Classical quantitative analysis	TRPV1 has no role in weight control, but has a role in stimulating the proinflammatory network that is central to hypertension	Marshall et al. (2013)
Diabetes, hyperlipidemia, and obesity	*In vivo*, olanzapine-induced metabolic syndrome in female BALB/c mice	Quantitative PCR	Hypothalamic TRPV1 mRNA increased	Singh et al. (2020)
Obesity	*In vivo*, HFD-induced obesity in male Wistar rats	qRT-PCR; Western blotting; Immunohistochemistry; Quantitative analyses	TRPV1 mRNA and protein increased in visceral adipose tissue	Cocci et al. (2021)
Hyperlipidemia and NAFLD	*In vivo*, high cholesterol and fat diet-induced hyperlipidemia in male Wister rats	Immunohistochemistry; Quantitative analyses	Hepatic TRPV1 increased	Harb et al. (2019)
Obesity	*In vivo*, HFD-induced obesity in male WT and TRPV1^−/−^ mice.*In vitro*, primary epididymal and subcutaneous preadipocytes	Western blotting; Immunoprecipitation, Quantitative RT-PCR; Intracellular Ca^2+^imaging; Quantitative analyses	TRPV1 mRNA and protein decreased in epididymal fat of WT-fed HFD; TRPV1 channel activity decreased in preadipocytes	Baskaran et al. (2016)
Obesity	*In vivo*, HFD-induced obesity in male WT and TRPV1^−/−^ mice	Western blot; Quantitative RT-PCR; Quantitative analyses	TRPV1 mRNA and protein decreased in epididymal fat	Krishnan et al. (2019)
Hyperlipidemia	*In vivo*, HFD-induced hyperlipidemia in male Sprague Dawley rats	Western blotting; quantitative PCR	Hepatic TRPV1 mRNA and protein decreased	Wang et al. (2020)
Atherosclerosis, dyslipidemia, and fatty liver disease	*In vivo*, male ApoE^−/−^, and ApoE^−/−^TRPV1^−/−^ mice fed high cholesterol and fat diet	Western blotting; Quantitative analyses	Hepatic HDL receptor (SR-BI) decreased in ApoE^−/−^TRPV1−/− mice compared to ApoE−/− mice; Serum levels of pro-inflammatory cytokines TNF-a, MCP-1, IL-6 and MIP-2 increased in ApoE^−/−^TRPV1^−/−^ mice	Wei et al. (2013)
Obesity, hypertension and hyperlipidemia	*In vivo*, high cholesterol and fat diet induced-metabolic syndrome in male Ossabaw pigs	Western immunoblot; Quantitative RT- PCR; Quantitative analyses; Immunohistochemistry	TRPV1 mRNA increased in artery, TRPV1 protein decreased in artery, TRPV1 decreased in endothelium of coronary arteries	Bratz et al. (2008)
NAFLD	*In vivo*, HFD-induced NAFLD in male C57BL/6 J wild-type and TRPV1 knockout mice; *In vitro*, HepG2 cell line exposed to free fatty acids	Semiquantitative conventional PCR; Immunoblotting; Quantitative analysis	Hepatic TRPV1 mRNA and protein decreased in both models	Li et al. (2013)
Dyslipidemia	*In vivo,* HFD induced dyslipidemia in Sprague Dawley rats	Western blotting; Quantitative analysis	Hepatic TRPV1 protein decreased	Gong et al. (2022)

## 6 Potential molecular mechanisms underlying TRPV1 regulation of lipid metabolism

### 6.1 Uncoupling proteins (UCPs)

Uncoupling proteins (UCPs) are inner mitochondrial membrane proteins that belong to the family of mitochondrial transporter proteins that play a role in lowering mitochondrial membrane potential, dissipation of metabolic energy as heat, maintenance of respiration, and prevention of reactive oxygen species (ROS) accumulation (Pierelli et al., 2017). UCPs decrease the membrane potential by acting as proton channels, leading to proton re-entry to the mitochondrial matrix from the intermembrane space and thus collapsing the proton gradient (Monteiro et al., 2021). They also maintain glucose homeostasis through stimulating insulin-mediated glucose uptake (Neschen et al., 2008). In mammals, five isotypes of UCPs have been characterized: UCP1-5. Among these isotypes, UCP2 plays a role in lipid metabolism, mitochondrial bioenergetics, oxidative stress, and apoptosis (Li et al., 2012; Pierelli et al., 2017). Li et al. (2012) have shown that capsaicin, an agonist for TRPV1, prevented HFD-induced fatty liver in mice through upregulation of hepatic UCP2 (Li et al., 2012). Moreover, dietary capsaicin induced browning of white adipose tissue to enhance energy expenditure and to prevent obesity through a signaling pathway that involves an increase in the expression of TRPV1 and its activity and an upregulation of UCP1. UCP1 has a crucial role in the regulation of brown fat thermogenesis (Baskaran et al., 2016). Likewise, activation of TRPV1 by monoacylglycerols and capsaicin was found to upregulate UCP1 and to suppress visceral fat accumulation (Iwasaki et al., 2011). Consistently, activation of TRPV1 expressed in the thoracic aorta vasculature inhibited the development of hypertension and vascular damage induced by HFD through upregulation of the metabolically important UCP1 (Schilling, et al., 2017). In addition, double-knockout of TRPV1 and UCP1 appeared to cause obesity and hypertension in mice, suggesting their crucial role in the prevention of obesity (Li et al., 2018; Li et al., 2022). Li et al. (2022) found a regulatory role for TRPV1 on LETM1, a mitochondrial inner membrane protein that mediates mitochondrial Ca^2+^ uptake and extrusion in a gradient-dependent manner, which was critical to restrain obesity and related hypertension. They found that UCP1 knock out in TRPV^−/−^ mice exacerbated obesity and produced more severe hypertension.

### 6.2 ATP-binding cassette (ABC) transporters

ATP-binding cassette (ABC) transporters are among the largest transport protein superfamilies with 49 members that are further divided into seven subfamilies (A to G) according to sequence similarity and domain organization of the members. Due to their ability to regulate lipid metabolism, ABC transporters have recently received significant attention in metabolic disorder therapy. ABC transporters maintain cholesterol homeostasis through participating in cholesterol uptake, biosynthesis, and storage (Ye et al., 2020). Evodiamine, a TRPV1 agonist, provides TRPV1-dependent atheroprotection through enhancing hepatic cholesterol clearance, as evidenced by upregulation of hepatic low-density lipoprotein receptor (LDLR), ABCG5, ABCG8 and cholesterol 7α-hydrolase (Wei et al., 2013). In addition, treatment with TRPV1 agonists facilitated cholesterol efflux by upregulation of ABCA1 and ABCG1 (Zhao et al., 2013).

### 6.3 Peroxisome proliferation-activated receptors (PPAR)

PPARs are ligand-activated transcription factors involved in the regulation of genes implicated in energy homeostasis. The three isotypes of PPARs identified, PPAR-α, PPAR-γ, and PPAR-δ, are encoded by different genes and they show different expression patterns and functions. PPAR-α controls the expression of catabolic genes and PPAR-γ controls genes involved in the storage of fatty acids whereas PPAR-δ is a key regulator in cholesterol metabolism and plays an atherogenic role in healthy humans (Skogsberg, 2003). The activation of PPAR-δ by agonists like GW501516 in dyslipidemic men provided beneficial effects on lipoprotein for it increased HDL cholesterol, decreased cholesteryl ester transfer protein activity and decreased plasma triglycerides and fatty acids (Ooi et al., 2011). Li et al. (2013) demonstrated that TRPV1 activation by dietary capsaicin upregulated PPAR-δ and hepatic hormone-sensitive lipase, thus promoting hepatic lipolysis without affecting lipogenesis. It was also found that TRPV1 activation prevented NAFLD through PPAR-δ-dependent autophagy enhancement in mice. Similarly, chronic dietary TRPV1 activation by capsaicin induced PPAR-δ expression to attenuate chronic high-salt-induced cardiac hypertrophy (Gao et al., 2014).

Luo et al. (2012) showed that PPAR-γ coactivator-1α (PGC1α), a transcriptional coactivator of nuclear receptors that regulate *β*-Oxidation of fatty acids, was upregulated by capsaicin-induced TRPV1 activation or genetic overexpression of TRPV1 in skeletal muscle in mice (Yazawa et al., 2010). Furthermore, trans-pellitorine, an alkamide found in *Piper nigrum,* has anti-adipogenic activity through indirect activation of TRPV1 and transient receptor potential ankyrin 1 (TRPA1), an effect that was blocked by specific inhibitors of TRPV1 and TRPA1. Treatment with trans-pellitorine during adipogenesis was found to be through reducing PPAR-γ expression at the gene and protein levels and decreasing the expression of the gene encoding fatty acid synthase, and these two effects are mediated *via* activation of TRPV1 and TRPA1 in 3T3-L1 cells (Lieder et al., 2017). Moreover, the development of hypertension and vascular damage in the thoracic aorta induced by HFD was prevented by activation of TRPV1 and this was accompanied by upregulation of PPAR-α, sirtuin-1 (central cellular metabolic sensor; SiRT-1), and PGC-1α (Schilling, et al., 2017).

The link of TRPV1 to PPAR-γ was stressed in further publications. For example, TRPV1 activation prevented HFD-induced obesity in mice, an effect that was associated with increased expression and deacetylation of PPAR-γ in the epididymal fat of these mice. Moreover, PPAR-γ and other thermogenic genes were enhanced by the overexpression of TRPV1 in cultured 3T3-L1 preadipocytes (Krishnan et al., 2019). Consistently, using dorsomorphin to inhibit AMPK and GW9662 to inhibit PPAR-γ, capsaicin was found to inhibit neutral lipid accumulation through upregulation of TRPV1 and PPAR-γ in HepG2 cells exposed to oleic acid, activation of AMPK, and inhibition of Akt/mTOR pathways thus regulating lipogenesis (Bort et al., 2019).

### 6.4 Sterol responsive element binding proteins (SREBPs)

Sterol responsive element binding proteins (SREBPs) are transcriptional factors that regulate lipid and cholesterol metabolism by controlling the expression of the genes related to biosynthesis and uptake of fatty acids, triglycerides, cholesterol, and phospholipids (Watanabe and Uesugi, 2013). When eugenol, a vanilloid compound that activates TRPV1 receptors, was applied to hepatocytes isolated from HFD-fed mice for 24 h, lipid content was markedly decreased, gene expression of SREBP1 was suppressed and its target enzymes were suppressed but those of lipolysis-related proteins were increased. CAMKK and AMPK and acetyl-CoA carboxylase were increased but that of p-mTOR was suppressed with 100 μM of eugenol (Jo et al., 2014). Capsaicin also reduced the neutral lipid content in HepG2 cells by inhibiting the AKT/mTOR pathway and SREBP-1c (Bort et al., 2019). Tart cherry supplements downregulated the HFD-induced expression of adipogenesis-related genes including SREBP-1c accompanied by down regulation of TRPV1 (Cocci et al., 2021). Similarly, zanthoxylum alkylamides ameliorated abnormal lipid metabolism in rats fed HFD through upregulation of TRPV1 and downregulation of SREBP-1c (Wang et al., 2020). It seems that the lipid lowering effect exerted by these plants products is attributed to modulation of TRPV1 that may be involved in the down regulation of SREBP-1c.

### 6.5 Hypoxia

Hypoxia is one of the factors that may contribute to metabolic disorders (Wei et al., 2020). Ischemia, an insufficient blood and oxygen supply to a tissue, may induce a reversible accumulation of free fatty acids which are stored mainly as triglycerides in lipid droplets in cells. The mechanism of hypoxia-induced metabolic changes in lipid metabolism is not well understood, however, it could be, in part, correlated with a reduction in mitochondrial NAD^+^ concentration in the absence of oxygen, thus inhibiting fatty acid-oxidation (Gimm et al., 2010). Indeed, it has been found that HFD induced hepatic TG accumulation and concomitant hypoxia. Conversely, hypoxia induced hepatic TG accumulation in mice and in cultured hepatocytes through inhibiting lipolytic and stimulating lipogenic genes expression. Therefore, lipotoxicity and hypoxia might work as reciprocal causation and both interact to promote the development of NAFLD (Zhang X. et al., 2020).

Hypoxia-inducible factor (HIF) system is a key regulator of a wide range of cellular and systemic responses to hypoxia and is found in all mammalian cells (Weidemann and Johnson, 2008). In hypoxic cardiomyocytes, HIF-1 was shown to participate in the accumulation of intracellular neutral lipids through inhibiting PPAR-α activity and reducing fatty acid utilization (Belanger et al., 2007). Furthermore, upregulation of HIF-2α activates lipid synthesis *via* the PI3K-AKT-mTOR pathway under hypoxic microenvironment (Chen et al., 2019).

Hypoxia was found to sensitize TRPV1 (Ristoiu et al., 2011). These authors reported that the activity, but not the expression, of TRPV1 was enhanced through activation of HIF-1a and PKCε under hypoxia. The amount of phosphorylated form of TRPV1 was increased when both native rat sensory neurons and human embryonic kidney-derived 293 cells expressing rat or human TRPV1 were exposed to hypoxia and high glucose. The phosphorylation of TRPV1 channels *via* a PKCε-dependent mechanism and HIF-1a hypoxia-dependent activation seems to be greatly involved during increased TRPV1 activity (Ristoiu et al., 2011). Wei et al. (2020) demonstrated that TRPV1 activation alleviates hypoxic injury in cardiomyocytes by activating the autophagy-lysosome pathway which involves the AMPK signaling pathway. They showed that the expression of TRPV1 was significantly increased under hypoxic compared to normoxic conditions. The expression of TRPV1 was further upregulated by capsaicin which improved the autophagy flux and protected cardiomyocytes from hypoxic damage (Wei et al., 2020). It is worthy to note that although hypoxia partly activated TRPV1 receptors it decreased their sensitivity to capsaicin (Kim et al., 2012). These contradictory effects were attributed to changes in ROS under hypoxia that might underlie the side-specific effects of ROS on TRPV1, being stimulatory on the intracellular side and inhibitory on the extracellular side. The interested reader would find an excellent discussion and a schematic model for the hypoxia-induced modulation of the TRPV1 activity in Kim et al. (2012).

## 7 Conclusion

TRPV1-mediated signaling is involved in the regulation of many cellular functions in both health and disease. Therefore, TRPV1 has received a great attention as a potential target to treat different disorders including inflammation, obesity, hyperlipidemia and other metabolic diseases. However, TRPV1 exhibits an ambiguous behavior with regard to its expression and activation and the results of many studies dealing with its role in lipid metabolism are contradictory. Many observations reported enhanced TRPV1 expression in metabolic disorders associated with hyperlipidemia while other observation reported suppression of TRPV1 expression in metabolic disorders. These contradictory aspects require precautions when data is interpreted and awaits reconciliation.

Although studying the role of TRPV1 in lipid metabolism and TRPV1-lipid interaction are complex and demanding, so far it helped deepening the knowledge about TRPV1 functioning and offered promising approaches for treatment of metabolic syndrome associated with hyperlipidemia. Therefore, further molecular investigations are needed to understand the exact role of TRPV1 in the regulation of lipid metabolism. In particular, the dynamics of TRPV1 expression should be more precisely determined in the desensitization and internalization processes which may affect the final outcome. Finally, the role of lipids and lipid mediators on TRPV1 receptor should be assessed to emphasize their interactions with TRPV1 and their modulating effect.
