# Supplementary material for: The interaction of TRPV1 and lipids: Insights into lipid metabolism
Source: Front Physiol. 2022 Dec 15;13:1066023. doi: 10.3389/fphys.2022.1066023 (PMC9797668; doi:10.3389/fphys.2022.1066023)
Supplement: Supplementary file 1 [file Image1.pdf]

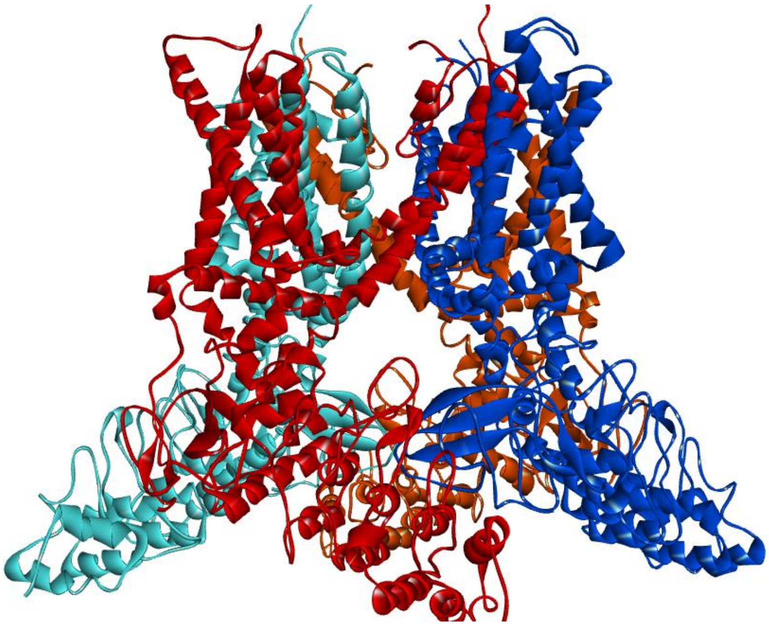

Supplementary Figure 1. Ribbon diagram of TRPV1 showing 4-fold symmetric architecture with each of the four identical subunits color-coded (polypeptide chains A-D: A is red, B is light blue, C is brown, and D is Dark blue). The solvent accessible surface area of TRPV1(PDB ID: 7LQZ). The figure was created by one of the authors using Biovia DS visualizer version 2017 after downloading PDB file (ID 7LQZ).
